# Do Prior Contraceptive Methods Impact Maternal Carriage in Patients with Hepatitis B?

**DOI:** 10.5812/kowsar.1735143X.774

**Published:** 2011-10-01

**Authors:** Terence T Lao, Oi Ka Chan, Stephen Sik Hung Suen, Tak Yeung Leung

**Affiliations:** 1 Department of Obstetrics and Gynaecology, Chinese University of Hong Kong, Hong Kong, China

**Keywords:** Contraceptive Agents, Hepatitis B, Pregnant Women

## Abstract

**Background:**

Hepatitis B virus (HBV) infection is highly endemic in many Asian countries.

**Objectives:**

We examined whether prior contraceptive methods and sexual behavioral factors impact maternal HBV carriage in an obstetric population.

**Patients and Methods:**

For this study, pregnant women were considered to be representative of the sexually active and fertile female population. Contraceptive methods used prior to the index pregnancy were examined in 1283 pregnant Chinese women attending an antenatal clinic using a self-administered questionnaire, and correlated with the maternal HBV status determined using routine antenatal screening.

**Results:**

In our study, 111 (8.7%) women were infected with HBV and there was no difference in the incidence of male condom usage between HBV-positive (88.3%) and HBV-negative (83.5%) women. No contraceptive method was associated with a reduced incidence of maternal HBV carriage, except for coitus interruptus. In multivariate analysis, only multiparity (adjusted odds ratio [aOR], 1.62) and more than 1 sexual partner (aOR, 1.57) were independent factors associated with maternal HBV carriage.

**Conclusions:**

Contraceptive use played only a minimal role in preventing sexual transmission of HBV infection within the sexually active female population in an endemic area.

## 1. Background

Hepatitis B infection is an infectious disease with over 350 million chronic carriers worldwide [1]. It is highly endemic in many Asian countries such as Hong Kong. Hepatitis B virus (HBV) infection can be acquired during embryonic development through vertical and perinatal transmission from a carrier mother, as well as by horizontal transmission through contact with blood and other body fluids. HBV infection can also be sexually transmitted in adults [2][3][4], particularly in high-risk groups such as homosexual males [5][6]. Among homosexual males, HBV is transmitted 8.6-fold more efficiently than HIV infection [6]. Furthermore, unprotected sexual exposure is an important mode of transmission in the Chinese population [7]. Controlling HBV infection is dependent on interrupting its transmission among individuals, and therefore failure to interrupt its transmission during sexual intercourse may influence the prevalence of HBV infection. Hong Kong is endemic in chronic HBV infection, with an overall prevalence of approximately 10% in the obstetric population [8]; thus, antenatal screening for hepatitis B surface antigen (HBsAg) status is performed routinely. A previous study showed that among the standard contraceptive methods available, the male condom is the most popularly used method [9]. Since condom usage has the additional effect of protecting against sexually transmitted diseases [10], the prevalence of HBV infection among the obstetric population may be influenced by the previous use of condoms before the index pregnancy.

## 2. Objectives

Since little information is available regarding the relationship between the use of contraceptive methods before pregnancy and HBV carriage diagnosed during the antenatal period in the obstetric population of an endemic region, we conducted a cross-sectional study on unselected pregnant women attending an antenatal clinic to elucidate the relationship between maternal HBV carriage, and the use of contraceptive methods as well as demographic, obstetric, and sexual behavioral factors.

## 3. Materials and Methods 

### 3.1. Patients and Study Procedures

A prospective, cross-sectional questionnaire-based survey to investigate health issues in the obstetric population was conducted in a university obstetric unit in Hong Kong over a 2-month period in 2008. Ethical approval was obtained from the hospital Institutional Review Board. Briefly, a total of 2000 questionnaires were distributed to consecutive Chinese women attending the antenatal clinic who were invited to participate in the study. Consenting women were asked to complete the questionnaire by themselves without advice or interference during their waiting time and to return the questionnaire to the medical staff at the time of antenatal examination. We limited the study to include Chinese women to eliminate the confounding effects of varying ethnicities and cultural backgrounds. Details regarding recruitment were described previously [11], and findings from different parts of the study have been reported previously [11][12][13]. Data regarding contraception and maternal hepatitis B status was the focus of this portion of the study. Confining the study to include Chinese women minimized the potential impact of cultural differences such as the attitude towards sexual behavior and the choice of contraceptive methods in the local female population of reproductive age. Questionnaires with incomplete answers concerning the contraception method used were excluded from the data. The survey instrument contained questions regarding the number of sexual partners of each respondent and the last contraceptive methods used before the index pregnancy. More than 1 choice could be selected for previously used contraceptive methods. The questionnaire also included information regarding sociodemographic factors and obstetric characteristics. Questionnaires with incomplete data on contraceptive methods used were excluded from final analysis. Hormonal injections, oral contraceptive pills (OCP), emergency contraception, and hormonal patches and implants were categorized as hormonal contraceptive methods, while the male condom, coitus interruptus, safety period, and spermicides were categorized as barrier contraceptive methods. Intrauterine contraceptive devices (IUCD) were analyzed as a separate group.

In Hong Kong, antenatal screening of maternal HBsAg status is routinely used to identify high-risk pregnancies for combined active-passive immunization to newborn infants who would otherwise receive only the hepatitis B vaccine at birth. Testing for hepatitis B surface antibody and hepatitis B e-antigen is not performed for those who tested negative for HBsAg. Therefore, the identification of maternal HBV infection in this study is dependent on a positive HBsAg status at routine antenatal screening, which is performed either at the Maternal and Child Health Centers or at the booking hospital. For subjects in this study, the results of the HBsAg screening were retrieved from an antenatal database. The relationship between HBV carriage and the use of each contraceptive method, as well as the use of hormonal, barrier, and IUCD methods, were analyzed.

### 3.2. Statistical Analysis

Statistical analysis was performed using the Statistical Package for Social Science (SPSS) version 17.0 (SPSS, Chicago, IL, USA). The distribution of continuous data was tested for normality. Continuous variables were compared using the t-test or Mann-Whitney U-test as appropriate. Categorical variables were compared using the chi-square test or Fisher exact test. The level of statistical significance was set at P < 0.05 (2-sided). Variables found to be significant in univariate analysis and all contraception methods were included in multivariate analysis. Logistic regression analysis was performed to delineate factors associated with hepatitis B status. Odds ratios (OR), adjusted odds ratios (aOR), and 95% confidence intervals (CI) were estimated.

## 4. Results

A total of 1697 questionnaires were collected from the recruited women during the study period, yielding a response rate of 84% for the entire study. Among these, 315 women had not used contraception and 52 women did not answer this question. Therefore, 1330 valid questionnaires were used for this study. Within this group, 47 did not proceed with further antenatal care in our hospital after attending the first-trimester combined screening program for Down's syndrome. Therefore, HBsAg status was determined only in 1283 women, of whom 111 (8.7%) were positive and 1172 (91.3%) were negative. There were no significant differences in HBsAg status between women who had used contraception (11.3%, P = 0.153) and those who did not respond to this question (5.1%, P = 0.768). In the study group, the mean age was 30.6 (standard deviation [SD], 4.7) years. Most were born in Hong Kong (775, 60.4%), were married or cohabiting (1189, 92.7%), and had resided in Hong Kong for at least 7 years (1001, 78.0%). There were 737 (57.4%) nulliparas and 545 (42.5%) multiparas. The 6 contraceptive methods used most frequently, in order of decreasing frequency, were the male condom (1077, 83.9%), safety period (301, 23.5%), coitus interruptus (274, 21.4%), OCP (147, 11.5%), IUCD (86, 6.7%), and hormonal injection (53, 4.1%).

The relationship between maternal HBsAg status, and contraceptive methods and obstetric and sexual behavioral factors is shown in Table 1. Maternal HBV carriage was significantly associated with a higher incidence of hormonal injection use (OR 2.26) and a lower incidence of coitus interruptus use (OR 0.51). In terms of obstetric and sexual behavioral factors, HBV carriage was associated with a higher incidence of multiparity (OR 1.54) and more than 1 sexual partner (OR 1.63). In the multivariate logistic regression analysis, which took into account all contraception methods, the use of hormonal injection (aOR 2.69, 95% CI 1.19-6.06, P = 0.017), multiparity (aOR, 1.61; 95% CI, 1.08-2.41; P = 0.021), and more than 1 sexual partner (aOR, 1.57; 95% CI, 1.05-2.35; P = 0.028), were independently associated with maternal HBV carriage, while use of coitus interruptus (aOR, 0.55; 95% CI, 0.30-1.01; P = 0.052) was just below the threshold for significance in the association with reduced risk of HBV carriage. To eliminate the confounding effects of parity and number of sexual partners in this study, each variable was stratified. For nulliparas, HBV carriage was associated with a higher incidence of using hormonal injection (OR, 5.35), IUCD (OR, 3.41) and a lower incidence of using coitus interruptus (OR, 0.41), while no significant association with these factors was observed for multiparas (Table 2). Similarly, for women who had more than 1 sexual partner, HBV carriage was associated with a higher incidence of using hormonal injection (OR, 3.54) and a lower incidence of using coitus interruptus (OR, 0.37), while no such associations were observed for those with a single sexual partner (Table 3).

**Table 1:** Use of Contraceptive Methods, and Obstetric and Sexual Behavioral Factors Stratified by Maternal HBsAg Status (n = 1283)

	**HBsAg**		**P value**	**OR a (95% CI a)**
	**Positive, No. (%) (n = 111)**	**Negative, No. (%) (n = 1172)**		
Contraceptive method				
Safety period	21 (18.9)	280 (23.9)	0.237	-
Coitus interruptus	14 (12.6)	260 (22.2)	0.019	0.51 (0.28–0.90)
Male condom	98 (88.3)	979 (83.5)	0.192	-
Oral contraceptive pills	11 (9.9)	136 (11.6)	0.592	-
Hormonal injection	9 (8.1)	44 (3.8)	0.041	2.26 (1.07–4.77)
Hormonal patch	1 (0.9)	19 (1.6)	1.000	-
Emergency contraception	3 (2.7)	31 (2.6)	1.000	-
IUCD a	8 (7.2)	78 (6.7)	0.824	-
Implant	1 (0.9)	8 (0.7)	0.558	-
Spermicides	0 (0)	8 (0.7)	1.000	-
Demographic, obstetric, and sexual-related factors				
Multiparity	58 (52.3)	487 (41.6)	0.030	1.54 (1.04–2.27)
Multi sexual partner, (n = 1181)	48 (48)	391 (36.2)	0.019	1.63 (1.08–2.46)
Used 2 or more contraception methods	37 (33.3)	430 (36.7)	0.483	-
Maternal age, mean ± SD	30.7 ± 4.7	30.5 ± 4.7	0.803	-

^a^ Abbreviation: CI, confidence interval ; IUCD, intrauterine contraceptive device ; OR, odds ratio

**Table 2:** Use of Contraceptive Methods Stratified by HBsAg and Parity Status (n = 1283)

	**Multiparas (n = 545)**		**P value**	**Nulliparas (n = 738)**		**P value**	**OR a (95% CI a)**
	**HBsAg + ve, % (n = 58)**	**HBsAg – ve, % (n = 487)**		**HBsAg + ve, % (n = 53)**	**HBsAg – ve, % (n = 685)**		
Contraceptive method							
Safety period	24.1	23.2	0.874	13.2	24.4	0.064	0.41 (0.17–0.97)
Coitus interrupts	13.8	19.9	0.264	11.3	23.8	0.037	-
Male condom	82.8	80.7	0.706	94.3	85.5	0.073	-
Oral contraceptive pills	5.2	11.5	0.143	15.1	11.7	0.462	5.35 (2.26–12.69)
Hormonal injection	1.7	4.5	0.496	15.1	3.2	0.001	-
Hormonal patch	0.0	1.4	1.000	1.9	1.8	1.000	-
Emergency contraception	1.7	1.8	1.000	3.8	3.2	0.688	3.41 (1.10–10.59)
IUCD a	6.9	12.7	0.198	7.5	2.3	0.049	
Implant	0.0	0.8	1.000	1.9	0.6	0.312	-
Spermicides	0.0	0.8	1.000	0.0	0.6	1.000	-
Sexual related factors							
Multi sexual partner, (n = 1181)	47.3	33.0	0.036	48.9	38.4	0.164	-
Used 2 or more contraception methods	27.6	35.7	0.219	39.6	37.4	0.751	-
Maternal age, mean ± SD	31.3 ± 4.2	31.9 ± 4.4	0.267	30.0 ± 5.1	29.6 ± 4.6	0.501	-

^a^ Abbreviation: CI, confidence interval ; IUCD, intrauterine contraceptive device ; OR, odds ratio

**Table 3:** Use of Contraceptive Methods by Stratified by HBsAg Status and Number of Sexual Partners (n = 1181)

	**Single Sexual Partner (n = 742)**		**P value**	**Multi Sexual Partner (n = 439)**		**P value**	**OR a (95% CIa)**
	**HBsAg + ve, % (n = 52)**	**HBsAg – ve, % (n = 690)**		**HBsAg + ve, % (n = 48)**	**HBsAg – ve, % (n = 391)**		
Contraceptive method							
Safety period	23.1	26.2	0.617	16.7	21.7	0.417	-
Coitus interrupts	13.5	21.3	0.179	10.4	23.8	0.036	0.37 (0.14–0.97)
Male condom	88.5	85.5	0.557	87.5	81.3	0.294	-
Oral contraceptive pills	3.8	9.4	0.217	16.7	15.1	0.774	-
Hormonal injection	3.8	3.2	0.682	14.6	4.6	0.012	3.54 (1.39–8.97)
Hormonal patch	1.9	1.2	0.482	0.0	2.3	0.606	-
Emergency contraception	1.9	2.6	1.000	4.2	2.8	0.643	-
IUCD a	7.7	6.4	0.767	8.3	7.4	0.773	-
Implant	1.9	0.7	0.354	0.0	0.5	1.000	-
Spermicides	0.0	0.7	1.000	0.0	0.5	1.000	-
Obstetric and sexual related factors							
Multiparity	55.8	43.8	0.093	54.2	38.1	0.032	1.92 (1.05–3.51)
Used 2 or more contraception methods	28.8	36.4	0.275	39.6	39.4	0.979	-
Maternal age, mean ± SD	30.8 ± 3.8	31.1 ± 4.5	0.739	29.8 ± 5.4	29.8 ± 4.7	0.989	-

^a^ Abbreviation: CI, confidence interval ; IUCD, Intrauterine contraceptive device ; OR, odds ratio

Factors associated with the use of IUCD, hormonal, and barrier methods were examined. In our study group, 86 (6.7%) women had used IUCD for contraception (7.2% vs. 6.7% for HBV-positive and HBV-negative respectively, P = 0.824), 184 (14.3%) had used hormonal methods (15.3% vs. 14.2%; P = 0.759), and 1144 (89.2%) had used barrier methods (92.8% vs. 88.8%; P = 0.198) as contraception. The frequency of these 3 methods used by women included in the study according to parity is shown in Table 4. For nulliparas, HBV carriage was associated with a higher incidence of using hormonal methods (OR, 2.18; 95% CI, 1.14-4.16) and IUCD (OR, 3.41; 95% CI, 1.10-10.60); a trend towards a lower incidence of hormonal method use was observed in multiparas (OR, 0.33; 95% CI, 0.99-1.07). Multivariate logistic regressions were performed to explore factors associated with maternal HBV carriage while adjusting for these categories of contraceptive methods. Multiparity (aOR, 1.62; 95% CI, 1.09-2.42; P = 0.017) and more than 1 sexual partner (aOR, 1.57; 95% CI, 1.05-2.34; P = 0.028) were the only independent factors associated with maternal HBV carriage, while no categories of contraceptive methods were observed to have an independent association.

**Table 4:** Association between Various Categories of Contraceptive Methods Stratified by Parity Status (n = 1283)

	**Multiparas (n = 545)**		**t value**	**OR a (95% CI a)**	**Nulliparas (n = 738)**		**P value**	**OR a (95% CI a)**
**HBsAg + ve, % (n = 58)**	**HBsAg - ve, % (n = 487)**			**HBsAg + ve, % (n = 53)**	**HBsAg - ve, % (n = 685)**
Any hormonal methods	5.2	14.4	0.052	0.33 (0.99–1.07)	26.4	14.2	0.016	2.18 (1.14–4.16)
Any barrier methods	89.7	85.8	0.110	-	96.2	90.9	0.159	-
IUCD a	6.9	12.7	0.198	-	7.5	2.3	0.049	3.41 (1.10–10.60)

^a^ Abbreviation: CI, confidence interval ; IUCD, Intrauterine contraceptive device ; OR, odds ratio

## 5. Discussion

A number of risk factors for HBV carriage have been identified in previous studies, including age, education level, history of jaundice, tattooing, presence of the HCV antibody, and a history of surgery [14][15][16][17]. However, there is no consensus regarding factors for HBV carriage in the obstetric population. A previous study conducted in Ghana among pregnant women showed that socio-demographic and medical characteristics were not associated with HBV carriage [18]. However, there have been no reports regarding the relationship between the use of contraceptive methods or obstetric and sexual behavioral factors, and HBV carriage in pregnant women. This is the first study to examine this issue, particularly among a single ethnic group in a highly endemic area.

According to the World Health Organization definition, Hong Kong is regarded as an endemic area for HBV infection. To control HBV infection in the population, a universal neonatal immunization program was introduced in 1988 [19]; the HBV vaccine has since been made available for all non-immunized individuals. However, the rate of maternal HBV infection has increased over the past 3 decades, from 6.6% in the 1970s [20] and 7.4% in the 1980s [21], to 10.0% in the 1990s and the most recent decade [8][19]. We suspect that the failure to prevent transmission during sexual intercourse, as well as inadequate knowledge and awareness regarding HBV transmission, is a major factor contributing to the persistent high rate of HBV infection [12]. In the present study, we focused on contraceptive methods used previously by pregnant women, taking factors such as parity and number of sexual partners into account. Other sociodemographic and medical characteristics, such as occupation, education level, and family income, were not included in the regression analysis, as they did not show a significant association with HBV carriage in univariate analysis. In our study, the incidence of condom usage among HBsAg-positive women (88.3%) did not significantly differ from that found among HBsAg-negative women (83.5%). On the other hand, maternal HBV carriage was associated with the use of hormonal injection (OR 2.26), and which was further increased for women with more than 1 sexual partner (OR 3.54) and for nulliparas (OR 5.35). In contrast, HBV carriage was associated with reduced usage of coitus interruptus (OR 0.51), and it was further reduced in those with more than 1 sexual partner (OR 0.37) and nulliparas (OR 0.41). Both contraceptive methods remained significant factors in the multivariate analysis after adjustment for parity and number of sexual partners.

We believe that, in a population endemic for HBV infection and with an extremely high usage of male condoms, the use of male condoms does not offer a protective effect against HBV transmission. Only methods that interrupt the transmission of seminal fluid, such as coitus interruptus, are protective against HBV transmission, an effect that is amplified in women with more than 1 sexual partner and nulliparas. In contrast, as hormonal injection is an extremely effective contraceptive method, women using this method were not at risk for becoming pregnant, and therefore tended to allow vaginal ejaculation, increasing the risk of acquiring HBV infection, an effect that was magnified in women with more than 1 sexual partner and in nulliparas. Although individual methods influenced the association with HBV carriage, various categories of contraceptive methods (IUCD, hormonal, and barrier methods) did not have a significant impact, and only multiparity and number of sexual partners remained independent factors for HBV carriage when these categories were analyzed together in the regression model. Our findings indicate that when HBV carriage is analyzed with respect to contraceptive methods, as well as with respect to social and sexual behavioral factors in the obstetric, sexually active population, the use of various contraceptive methods played only a minimal role in facilitating or preventing the sexual transmission of HBV infection in an endemic area. There were some limitations to our study. Although our center is one of the largest obstetric units in our community with 6000 deliveries per year, the sample studied may not have been representative of the entire obstetric population in Hong Kong. Some women are managed in the private sector as suggested by the subjects excluded from this study. Furthermore, although the women were asked to indicate previously used contraceptive methods, detailed information regarding which method was predominantly used when more than 1 method was used could not be obtained. Other risk factors for HBV carriage, such as history of jaundice, presence of the hepatitis C virus antibody, and a history of surgery, were not included on the questionnaire. A potential confounding factor in this study was that our women may not have truthfully disclosed or answered sensitive items about sexual behavioral history, particularly when accompanied by their husbands or partners, thus resulting in unreliable responses. To exclude this confounding factor, we compared responses regarding the number of sexual partners from those attended the antenatal visit alone and those accompanied by others. There were no statistically significant differences between these 2 groups. Nevertheless, the findings of this study indicated that in highly endemic communities, no contraceptive method is associated with reduced incidence of maternal HBV carriage, except for coitus interruptus. The apparent protective effect of this method on maternal HBV carriage, particularly in nulliparas and women with more than 1 sexual partner, should be explored in further studies, as this method could be recommended to HBV-naïve women pending the establishment of HBV immunoprotection following vaccination.

## References

[1] World Health Organization. Hepatitis B. (Fact sheet no. 204). Geneva, Switzerland: WHO. [updated 2008].

[2] Workowski KA, Berman SM (2006). Sexually transmitted diseases treatment guidelines, 2006.. MMWR Recomm Rep.

[3] Atkins M, Nolan M (2005). Sexual transmission of hepatitis B.. Curr Opin Infect Dis.

[4] Ko YC, Yen YY, Yeh SM, Lan SJ (1989). Female to male transmission of hepatitis B virus between Chinese spouses.. J Med Virol.

[5] Russi JC, Serra M, Viñoles J, Pérez MT, Ruchansky D, Alonso G, Sanchez JL, Russell KL, Montano SM, Negrete M, Weissenbacher M (2003). Sexual transmission of hepatitis B virus, hepatitis C virus, and human immunodeficiency virus type 1 infections among male transvestite comercial sex workers in Montevideo, Uruguay.. Am J Trop Med Hyg.

[6] Kingsley LA, Rinaldo CR, Jr, Lyter DW, Valdiserri RO, Belle SH, Ho M (1990). Sexual transmission efficiency of hepatitis B virus and human immunodeficiency virus among homosexual men.. JAMA.

[7] Yan YX, Gao YQ, Sun X, Wang W, Huang XJ, Zhang T, Li M, Zang CP, Li ZC, Wu H (2011). Prevalence of hepatitis C virus and hepatitis B virus infections in HIV-positive Chinese patients.. Epidemiol Infect.

[8] Suen SS, Lao TT, Sahota DS, Lau TK, Leung TY (2010). Implications of the relationship between maternal age and parity with hepatitis B carrier status in a high endemicity area.. J Viral Hepat.

[9] The Family Planning Association of Hong Kong. (2009). Annual report 2008-2009.. Hong Kong.

[10] World Health Organization. Department of Reproductive Health and Research (WHO/RHR) and Johns Hopkins Bloomberg School of Public Health/Center for Communication Programs (CCP), INFO Project. Family Planning: A Global Handbook for Providers. Baltimore and Geneva:.

[11] Chan OK, Suen SS, Lao TT, Leung VK, Yeung SW, Leung TY (2009). Determinants of hepatitis B vaccine uptake among pregnant Chinese women in Hong Kong.. Int J Gynaecol Obstet.

[12] Chan OK, Lao TT, Suen SS, Lau TK, Leung TY (2010). Knowledge on hepatitis B infection among pregnant women in a high endemicity area.. Patient Educ Couns.

[13] Chan OK, Lao TT, Suen SS, Lau TK, Leung TY (2011). Correlation between maternal hepatitis B surface antigen carrier status with social, medical and family factors in an endemic area: have we overlooked something?. Infection.

[14] Butler TG, Dolan KA, Ferson MJ, McGuinness LM, Brown PR, Robertson PW (1997). Hepatitis B and C in New South Wales prisons: prevalence and risk factors.. Med J Aust.

[15] Luksamijarulkul P, Mooktaragosa A, Luksamijarulkul S (2002). Risk factors for hepatitis B surface antigen positivity among pregnant women.. J Med Assoc Thai.

[16] Al Awaidy S, Abu-Elyazeed R, Al Hosani H, Al Mulla A, Al Busaiedy S, Al Amiry A, Farah Z, Al Marrie A, Bock HL, Al-Shaar I, Shah S (2006). Sero-epidemiology of hepatitis B infection in pregnant women in Oman, Qatar and the United Arab Emirates.. J Infect.

[17] Duong TH, Nguyen PH, Henley K, Peters M (2009). Risk factors for hepatitis B infection in rural Vietnam.. Asian Pac J Cancer Prev.

[18] Damale NK, Lassey AT, Bekoe V (2005). Hepatitis B virus seroprevalence among parturients in Accra, Ghana.. Int J Gynaecol Obstet.

[19] Kwan LC, Ho YY, Lee SS (1997). The declining HBsAg carriage rate in pregnant women in Hong Kong.. Epidemiol Infect.

[20] Lee AK, Ip HM, Wong VC (1978). Mechanisms of maternal-fetal transmission of hepatitis B virus.. J Infect Dis.

[21] Wong VC, Ip HM, Reesink HW, Lelie PN, Reerink-Brongers EE, Yeung CY, Ma HK (1984). Prevention of the HBsAg carrier state in newborn infants of mothers who are chronic carriers of HBsAg and HBeAg by administration of hepatitis-B vaccine and hepatitis-B immunoglobulin. Double-blind randomised placebo-controlled study.. Lancet.

